# The dysregulation of innate immunity by *Porphyromonas gingivalis* in the etiology of Alzheimer's disease

**DOI:** 10.1111/joim.70060

**Published:** 2025-12-22

**Authors:** Annelise E. Barron, Jennifer S. Lin, Mark I. Ryder, Peter Bergman

**Affiliations:** ^1^ Department of Bioengineering Schools of Medicine and of Engineering Stanford University Stanford California USA; ^2^ Department of Laboratory Medicine Division of Clinical Immunology Karolinska Institutet Stockholm Sweden; ^3^ Department of Clinical Immunology and Transfusion Medicine Karolinska University Hospital Stockholm Sweden

**Keywords:** antimicrobial peptides, cathelicidin, herpesviruses, host defense peptides, HSV‐1, human cytomegalovirus (HCMV), LL‐37, *Porphyromonas gingivalis*

## Abstract

The etiology of Alzheimer's disease (AD) remains under active debate. In this perspective, we explore the hypothesis that a primarily infection‐caused chronic dysregulation and weakening of human innate immunity via the underexpression, degradation, and inactivation of innate immune proteins necessary for direct antimicrobial effects and regulation of host defense and autophagy could lead to AD. Key evidence relates to the fact that important innate immune proteins such as LL‐37—which can bind Aβ and block amyloid formation—as well as Apolipoprotein E, antiviral interferons, and TNF‐α can be degraded and deactivated by enzymes produced by the common oral anaerobic pathogen *Porphyromonas gingivalis* (*Pg*). *Pg* produces numerous virulence factors; of particular importance for AD are *Pg*’s gingipain cysteine proteases. Deleterious effects of chronic *Pg* infection and gingipains include a systemic downregulation and paralysis of the interferon response, particularly the antiviral interferon‐lambda response, which enables replication of endemic herpesviruses. The result is a chronic, low‐level viral infectious assault on gut, nerves, and brain causing the production of Aβ antimicrobial peptides, accumulation of Aβ plaques, phosphorylation of Tau, progressive neuroinflammation, and neurodegeneration. The resultant innate immune system dysregulation, as an AD etiology, ties together the well‐known amyloid cascade hypothesis and the infectious theory of AD into a unified explanation of the pathology and cause of AD. If this theory holds true, it suggests preventative approaches: (1) test for and eradicate *Pg* from oral flora, and/or directly deactivate the gingipains; and (2) reduce Herpesvirus exacerbations by the use of antiviral drugs and/or vaccines (e.g., Bacillus Calmette–Guérin).

## Graphical abstract

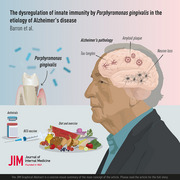

**Abbreviations**
Aβ: β‐amyloid
AD: Alzheimer's disease
ApoE: Apolipoprotein E
BBB: blood–brain barrier
*Ca*
: *Candida albicans*
HCMV: human cytomegalovirus
HSV‐1: herpes simplex virus 1
*Pg*
: *Porphyromonas gingivalis*

## Introduction

**Fig. 1:**
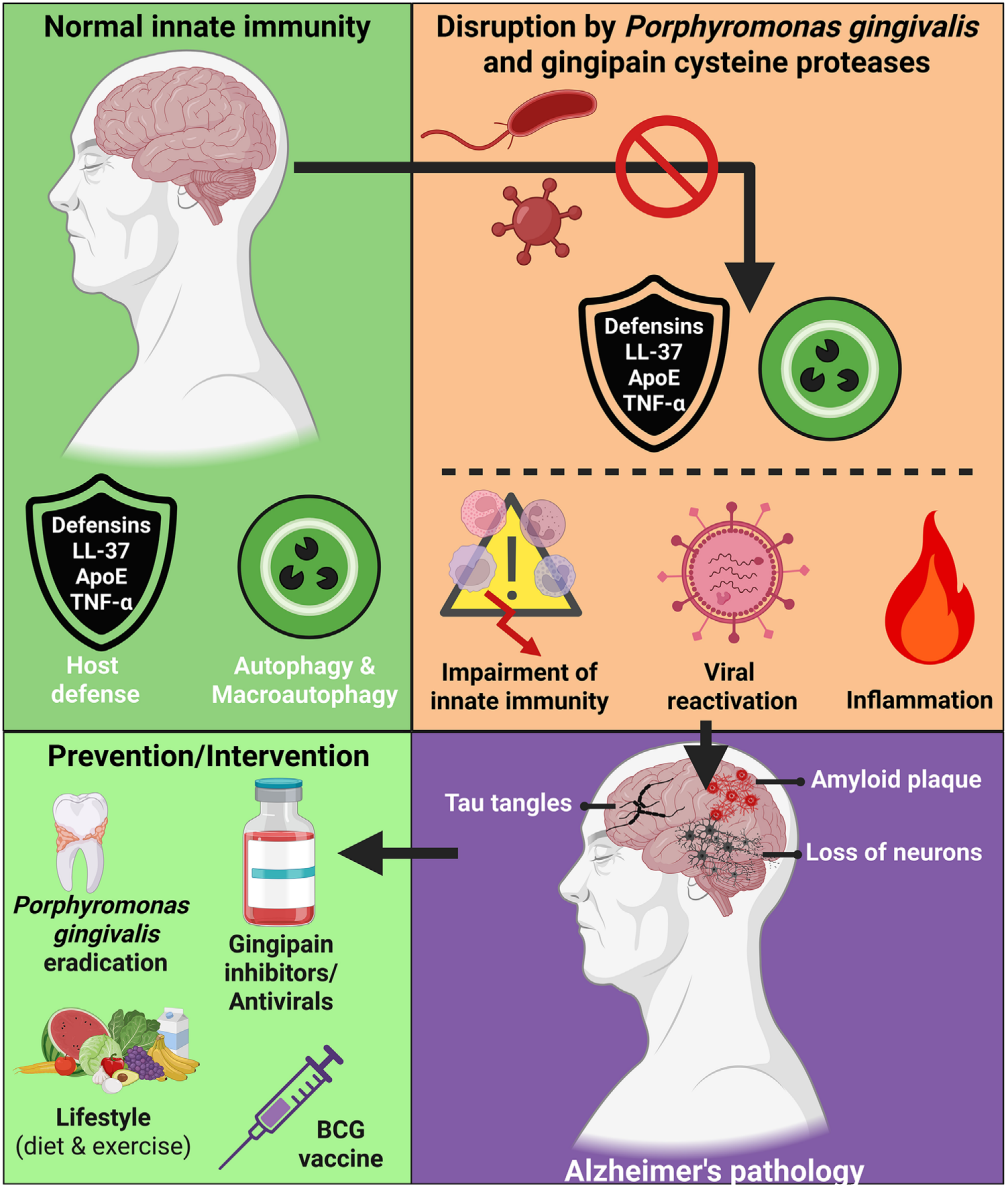
**Dysregulation of innate immunity as a potential cause of Alzheimer's disease (AD)**. This hypothesis unifies the amyloid cascade and infectious theories of AD through the lens of chronic innate immune system dysregulation and points to new prevention and treatment strategies for AD. BCG, Bacillus Calmette–Guérin. Source: Created with BioRender.

In this review, we will discuss a novel hypothesis that certain chronic, undiagnosed, and typically unperceived bacterial/viral coinfections can trigger the pathogenesis of Alzheimer's disease (AD) (Fig. 1). We build this hypothesis on several pieces of evidence, which are summarized briefly in the next few paragraphs and then discussed in more detail and with full referencing below that. First, although β‐amyloid (Aβ)‐plaque‐induced inflammation has typically been proposed as a “cause” of AD, it makes more sense that Aβ production and inflammation are downstream effects of infection. Indeed, it has been shown that infections can lead to Aβ plaque accumulation, and Aβ is known to be an antimicrobial peptide. Second, innate immune effectors—including Apolipoprotein E (ApoE), the interferons, and LL‐37 (another antimicrobial peptide, which can bind to and prevent Aβ fibril formation [1])—can be degraded by gingipain proteases released by the anaerobic bacterium *Porphyromonas gingivalis* (*Pg*), leaving the body vulnerable to infection by neurotropic herpesviruses, particularly to human cytomegalovirus (HCMV) and herpes simplex 1 (HSV‐1), both endemic in the human population and rarely treated. Third, it should be noted that other anaerobic oral pathogens can produce deleterious virulence factors and may have negative systemic effects. However, *Pg* is the most common and most important immune‐dysregulating pathogen. Fourth, *Pg*’s survival has been shown to be supported strongly by the polymorphic fungus *Candida albicans* (*Ca*)—with which *Pg* forms dual biofilms—providing a possible role for chronically elevated blood glucose in supporting *Pg* and AD's pathogenic processes and one potential reason why low‐carbohydrate “ketogenic” diets can have profound salutary effects on mental health and many other conditions. Finally, there are many neurotropic viruses that will replicate more freely when human interferon pathways are disrupted and downregulated by *Pg* gingipains or other bacterial virulence factors. This signifies that the landscape of potential coinfections that can lead to human cognitive changes and deficits is likely to be quite complex, and careful studies in human populations must be done to shed light on these important co‐infectious mechanisms of disease.

The good news is that if this hypothesis of causation by bacterial/viral (and possibly fungal) coinfection and resultant innate immune system dysregulation and inflammation holds true, then it immediately opens up new pathways for a multitude of diagnostic, preventive, and treatment strategies for cognitive loss and dementia, offering new, outsized potential benefits for people worldwide, including young people as well as patients with AD.

### Exploring a new bacterial/viral coinfection and innate immune system dysregulation hypothesis for the etiology of AD pathogenesis

We posit that the true cause of AD is the chronic dysregulation and weakening of human innate immunity as the result of bacterial/viral coinfection, accompanied by the underexpression, degradation, and/or inactivation of key innate immune proteins that are necessary for effective host defense and autophagy. Two of these innate immune proteins are ApoE (alleles 3, 4) and antimicrobial peptide LL‐37. Abolishment of the normal direct antimicrobial as well as the essential immunomodulatory activities of ApoE and LL‐37, which can be caused by the gingipain virulence factors of *Pg*, cripple the brain's innate immune system. As will be discussed below, *Pg* gingipains also dysregulate interferons and antiviral immunity, allowing neurotropic viruses to attack the nerves and brain. In the presence of chronic infections, there occurs a progressive accumulation of neuroinflammatory Aβ plaques and fibrils. At the same time, the innate immune system becomes dysregulated by *Pg* gingipains and has reduced ability to combat pathogens (within the oral cavity and the gut, nerves, and brain) or to kill infected cells (because intact ApoE protein is necessary to activate the complement cascade [2]). Unchecked entrenchment and progress of such coinfections can lead to neuroinflammation and neurodegeneration, the hallmarks of AD. Here, we tie together the amyloid cascade hypothesis and the infectious theory of AD into a single unified theory for the pathology and cause of AD.

In the context of this theory, late‐onset AD may be considered to result from a progressive accumulation of noxious inflammatory processes or factors in the brain, fueled by anaerobic bacterial and viral infectious agents that colonize and infect the oral cavity (and gut, and nerves) and can spread systemically. Local neuroinflammation may continue at a low level throughout life with little negative effect. However, this inflammation can be exacerbated and perpetuated by repeated reactivation of infection (particularly chronic herpesvirus infections) combined with other insults such as the deactivation or dysregulation of natural host defense proteins or age‐related insults such as immune cell senescence, or an acute inflammatory response resulting in unbalanced production or improper signaling by inflammatory cytokines, such as TNF‐α. Consequently, the inability to fully clear a chronic *Pg* infection can lead in time to chronic viral replication, neuroinflammation, and brain tissue damage. The relative balance of microglia, astrocytes, and neurons may be altered, and enhanced neuroinflammatory processes may then disrupt the blood–brain barrier (BBB). These mediators induce peripheral inflammation and then can return to further stimulate local neuroinflammation [3, 4, 5].

### Challenging the prevailing amyloid cascade hypothesis

The *true cause* of a human disease must be *necessary and sufficient* to produce the disease, and the removal of this cause should reverse the disease, provided that tissue degradation is not too extensive already [6].

The amyloid cascade hypothesis of AD [7] recognizes the accumulation of Aβ over time, which correlates in many individuals with a loss of cognitive function and the failure of the immune system to eliminate these accumulated Aβ plaques. The amyloid cascade theory suggests that as Aβ‐based plaques become more abundant, they are the cause of chronic neuroinflammation, which then triggers the intraneuronal accumulation of Tau proteins in a hyperphosphorylated form, compromising neuronal function. These changes ultimately result in neuronal death [7]. In the relatively rarer cases of early onset AD (5%–7% of Alzheimer's cases), various familial genetic mutations can lead to rapid accumulation of Aβ, with early onset of dementia often occurring in the late 40s or 50s of a patient's life [8]. However, there is not, at this time, any widely accepted explanation or cause for why there is an increased accumulation of Aβ in late‐onset AD, which is by far the more prevalent type of dementia (∼93%–95%).

It has become apparent that increases in Aβ production, or decreases in its elimination, may in fact be due to a physiological need for the peptide and its fibrils and plaques [9]. Initially, there were many diverse hypotheses for Aβ’s functions: It was put forward that Aβ activates kinase enzymes [10], protects against oxidative stress [11], regulates cholesterol transport [12], or functions as a transcription factor [13]. However, it was later discovered that Aβ is also an antimicrobial peptide [14, 15, 16, 17] (also called a host defense peptide [18]; or an innate immune effector). Interestingly, the amino acid sequence of the Aβ peptide is strongly conserved in many mammalian species, supporting the hypothesis that it fulfills an important and central function [19].

It was demonstrated in a series of in vitro experiments that Aβ could kill bacteria and fungi [14], and that influenza virus was inactivated by Aβ [20]. Notably, Aβ can also inactivate HSV‐1 [15], a virus that can latently colonize neural ganglia. Importantly, it was also shown that Aβ synthesis by neurons is stimulated by HSV‐1 infection [16]. It was found that the Aβ produced by this stimulation prevents HSV‐1 from killing neurons for at least 5 days, whereas epithelial cell cultures that do not make Aβ were completely destroyed within 24 h of HSV‐1 infection [16].

The reliance of the protection of the neurons on local physiological‐level production of Aβ was then further demonstrated by treating infected neuron cultures with a BACE‐1 inhibitor, which prevents Aβ production [16, 21]. Without Aβ production, the neuronal cells were destroyed. Moreover, Aβ not only prevents viral infection, but transgenic mice expressing human Aβ were shown to be protected from an intracerebral bacterial injection of *Salmonella typhimurium* [22], which otherwise would be lethal. Combined, based on these in vitro and in vivo experiments, there is strong evidence that Aβ is protective against infection.

As Aβ is a bona fide antimicrobial peptide that has proven in vivo effects at physiological concentrations, preventing late‐onset AD by inhibiting Aβ synthesis—a reduction of Aβ—would be expected to lead sometimes to deleterious effects. Indeed, 450+ clinical trials failed to reveal a single pharmaceutical product that fully prevents AD by blunting Aβ’s synthesis or by facilitating Aβ’s clearance [7]. The recent FDA approval of antibody drugs that drive the clearance of Aβ from the brain and body—moderately slowing the progression of AD at some risk of brain swelling and bleeding—has not yet resulted in widespread use of these drugs, which are seen to offer a marginal benefit along with significant risks [23, 24]. As Aβ synthesis is stimulated by HSV‐1 (and other pathogens) or their products, one way to lower Aβ synthesis is to reduce the presence of the stimulating pathogens. Hence, identification of these pathogens and the prevention or treatment of chronic, often undiagnosed and unperceived infections could potentially provide an important way to prevent or mitigate AD pathogenesis.

### Apolipoprotein E (ApoE)

ApoE powerfully modulates the risk for AD [25, 26] and has recently been found to play an important role in innate immunity during bacterial infections by countering bacterial endotoxins, with ApoE‐ε4 having a greater effect in increasing AD risk than ApoE‐ε3 [27]. Studies have shown that ApoE exhibits antibacterial activity, particularly against Gram‐negative bacteria [28]. Additionally, active ApoE is central to the resolution of infection and inflammation, because it plays an important role in activating the classical complement cascade via binding to C1q [29]. Importantly, emerging research has shown that gingipains from *Pg* can degrade ApoE, potentially not only disrupting innate immunity against bacterial infections, but also ApoE's function in the central nervous system, maintaining neuronal synapses [30].

### ApoE isoforms are differentially fragmented by gingipains from *Porphyromonas gingivalis*

**Fig. 2:**
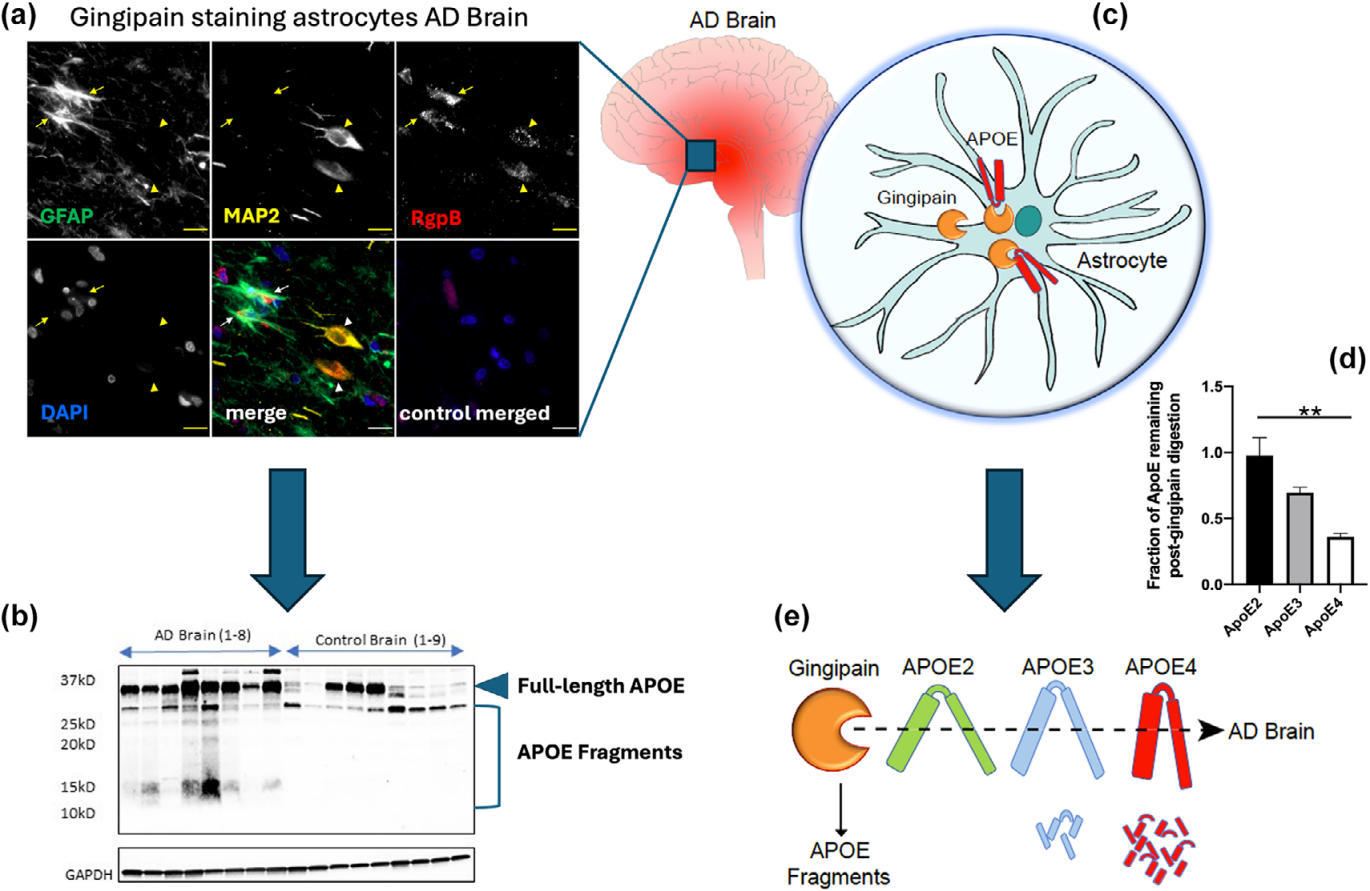
**Gingipains identified in brains of patients with Alzheimer's disease (AD) differentially fragment Apolipoprotein E (ApoE) proteins**. (a) RgpB gingipain associates with astrocytes and neurons in AD brain using immunofluorescence. (b) AD brains, but not control brains, demonstrate low‐molecular‐weight fragments of ApoE on a Western blot. (c) Graphic of gingipains inside of an astrocyte interacting with ApoE. (d) Data showing that when mouse brains containing human ApoE isoforms are incubated with gingipains, ApoE2 is the most resistant to gingipain digestion, followed by ApoE3, with ApoE4 the most susceptible to gingipain digestion. (e) Graphic of the differential fragmentation of ApoE by gingipains. Source: Adapted from [31]. BioRender used for (c) and (e).

Fragmented ApoE has been found in the brains of patients with AD in the presence of gingipains, the potent virulence factors released by the oral pathobiont *Pg* [31]. These Arg/Lys endopeptidases include lysine‐gingipain (Kgp), arginine‐gingipain A (RgpA), and arginine‐gingipain B (RgpB). Fig. 2 shows the presence of RgpB antigens colocalizing with astrocytes and neurons in AD brain tissue (Fig. 2a). Astrocytes are the main producers of ApoE in the brain, and ApoE is critical in the maintenance of synapses in neurons. Low‐molecular weight ApoE proteolytic cleavage products are observed in brain tissue samples of patients with AD, but not in control samples (Fig. 2b) [31]. Furthermore, in vitro experiments have demonstrated that ApoE‐ε4 is cleaved preferentially compared to ApoE‐ε3 and ApoE‐ε2 by *Pg*’s gingipains (Fig. 2d,e). This enhanced degradation of ApoE‐ε4 may explain why carriers of two ε4 alleles of ApoE have a significantly greater risk (up to 15x) of developing AD [25].

### Chronic periodontitis: linking chronic infection to AD's hallmark inflammation

Chronic periodontitis is associated with an increase in cognitive decline in AD, independent of baseline cognitive state [32, 33, 34]. It has been suggested [35] that *Pg* may be the key deleterious microorganism inside the mouth, and this hypothesis was strongly corroborated in 2019 [36]. Indeed, *Pg* and its virulence factors (gingipains) have been found in the brains of patients with AD, where the AD diagnosis correlates well with gingipain load. *Pg* DNA has also been found in the cerebrospinal fluid of patients with AD [36]. Additionally, oral *Pg* infection has been shown to downregulate the interferon response, both in mouse models of infection and in human periodontitis patients, resulting in “a broad paralysis” of the interferon response and, specifically, reduced antiviral immunity provided by interferon‐lambda (IFN‐λ) [37]. More recently, it was shown that *Pg* significantly increases HSV‐1 infection due to impairment of the interferon response. Proteolytic modifications to major signaling components of the interferon response were found to be catalyzed by Kgp gingipain [38]. Additionally, *Pg* infection was observed to promote reactivation of HSV‐1 in neuronal cells, although via an IFN‐independent mechanism.

Here, it is notable that Aβ peptide itself has anti‐*Pg* activity [36, 39]. Moreover, mice dosed orally with *Pg* were shown to develop neuroinflammation and the key hallmark lesions of late‐onset AD (i.e., Aβ plaques and phospho‐Tau neurofibrillary tangles), resulting in the death of 50% of the hippocampal neurons [40]. DNA from *Pg* has also been detected in human brain biopsies [36], along with *Pg* virulence factors, including the gingipain proteins.

Thus, chronic *Pg* infection may leave the brain, body, and neuronal networks (e.g., vagal nerve and trigeminal nerves) much more vulnerable to invasion by neurotropic viruses, especially those in the family of eight herpesviruses that are endemic in the human population. Herpesviruses can use the nerves as a pathway to reach the brain, and *within* a neuron, of course, there is no BBB. The trigeminal nerve complex enters the brainstem on the ventrolateral side of the pons, whereas its nuclear complex is distributed across multiple regions of the brainstem, including both the midbrain and pons.

### *Pg* virulence factors

As noted above, *Pg* produces enzymatic toxins [41] called gingipains, but also other endopeptidases, fimbriae, hemagglutinins, and bacterial lipopolysaccharide (LPS) [42]. The gingipains induce sustained chronic inflammation, inhibition of normal neutrophil functions [43], and activation of complement at low gingipain concentrations, but degradation of complement factors at higher gingipain concentrations [44]. In addition, gingipains degrade bioactive proteins (including host defense peptides [e.g., LL‐37], which are generally cationic with many arginine and lysine residues) [18]; cause degradation of immunoglobulins A and G and collagenase [45]; deregulate coagulation and the kinin cascade [46]; and disrupt signaling networks that control inflammatory processes [47].

**Fig. 3:**
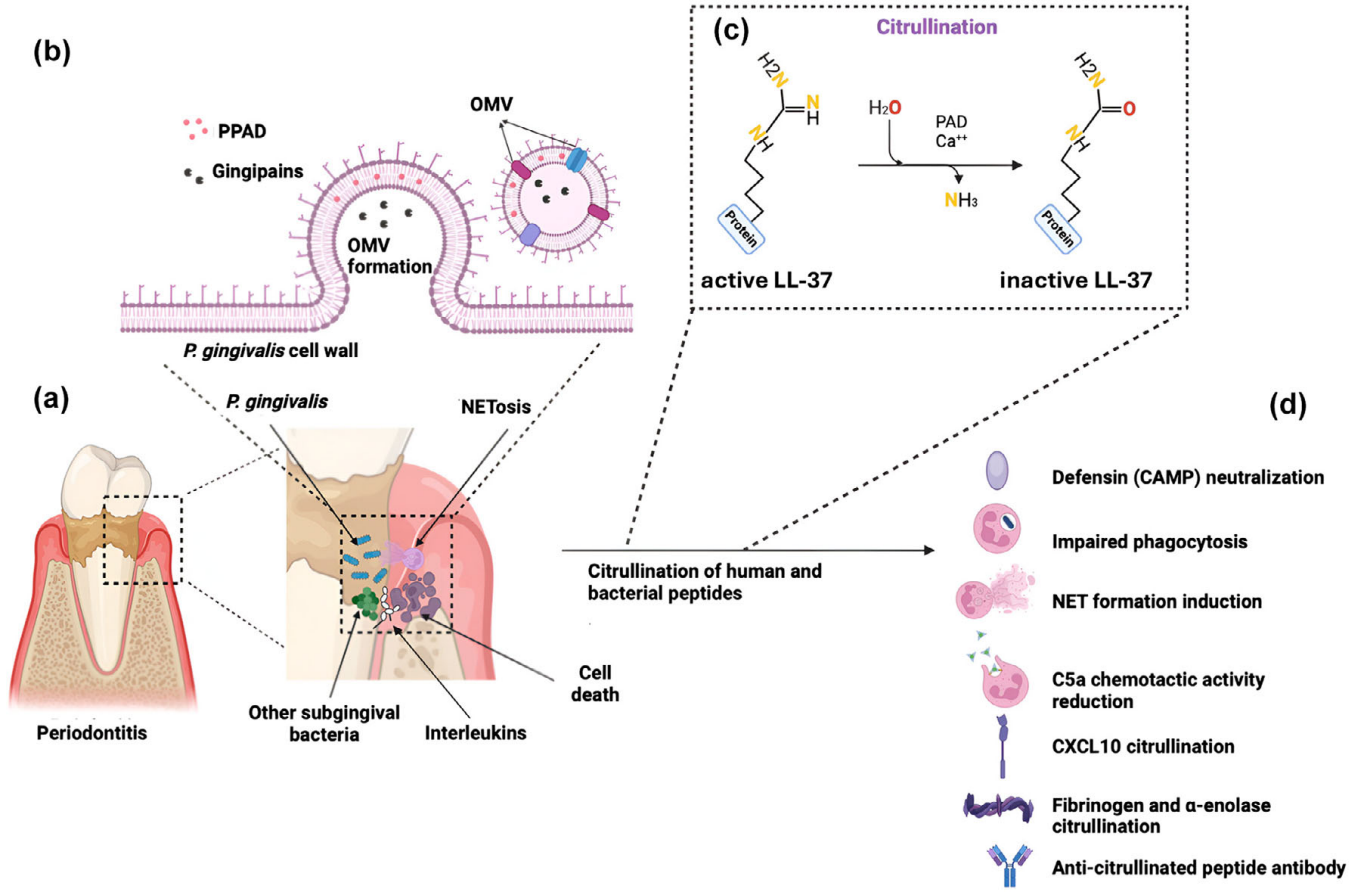
**Citrullination of LL‐37 by Porphyromonas gingivalis (Pg) peptidylarginine deiminase (PPAD) enzyme inhibits activity**. (a) Tooth with diseased periodontal pocket containing Pg bacteria. (b) Formation of Pg outer membrane vesicles (OMVs) containing gingipains and Pg PPAD. (c) PPAD citrullination of LL‐37 destroys its function. (d) List of immune proteins citrullinated by PPAD. Source: Adapted from [167]. Original image created with BioRender.

*Pg* also produces a highly active peptidylarginine deiminase (PPAD), an enzymatic virulence factor that converts exposed arginine side chains to citrulline moieties (see Fig. 3). Interestingly, AD hippocampi show an abnormal accumulation of citrullinated proteins [48].

*Pg*’s gingipains have been found to be neurotoxic both in vivo and in vitro and to exert detrimental effects on the Tau protein, which is required for normal neuronal function. Inhibitors have been designed to target these gingipains [36]. The use of these inhibitors was found to reduce the bacterial load of an established *Pg* brain infection, block Aβ42 production, reduce neuroinflammation, and rescue neurons in the hippocampus, thus demonstrating that gingipain inhibitors may be valuable future therapeutics for treating *Pg* brain colonization and neurodegeneration in AD [36].

### Human cathelicidin LL‐37: a missing puzzle piece in understanding AD pathogenesis

The cathelicidin peptide LL‐37, a human host defense/antimicrobial peptide, exhibits direct anti‐amyloidogenic activity in vitro by blocking Aβ oligomerization and could therefore play a key role in the development of AD [1]. Of course, LL‐37 is a broadly antimicrobial (antibacterial, antifungal, antiviral) peptide, as discussed in detail below [49]. On that basis, lower LL‐37 levels would be expected to allow infectious agents to replicate in human tissues, including possibly the brain. This infectious insult triggers an Aβ antimicrobial response that could lead to the formation of neuroinflammatory oligomers, fibrils, and plaques that dysregulate both innate and adaptive immunity responses.

Low levels of LL‐37 may arise in two different ways: (1) by *underexpression*, which can occur if a person has very low serum vitamin D3, vitamin A (or other RXRα agonists), and/or butyrate or fails to exercise (see below); or (2) by *degradation and deactivation*, which can occur after oral pathogens, especially *Pg*, are established below the gum line as biofilms and subsequently release a host of enzymatic virulence factors that can degrade and inactivate the LL‐37 peptide as well as other proteins such as TNF‐α, IL‐6, interferons, and ApoE. It may occur in many cases that LL‐37 is simultaneously underexpressed and also subject to degradation by microbial enzymes, resulting in very low levels of active peptide. This would present a worst‐case scenario, because LL‐37 also helps to regulate the integrity of the BBB [50, 51].

### LL‐37 is ancient and multifunctional

The antimicrobial peptide LL‐37 is a centrally important human host defense peptide and is the sole human cathelicidin [49, 52]. On an evolutionary basis, the family of cathelicidin proteins is extremely ancient: They occur even in the jawless hagfish [53], which arose over 300 million years ago. LL‐37 is encoded by the CAMP gene and is unique in the human proteome. Cathelicidins are also one‐of‐a‐kind within primates—including both old and new world monkeys [54]—and in mouse and rat proteomes (genes: *cramp and rcramp*, respectively) [49]. Uniquely within humans, monkeys, and apes, the expression of LL‐37 is vitamin D3‐dependent [54]. In addition to vitamin D3, a retinoid (or other RXRα agonist) such as vitamin A or docosahexaenoic acid (DHA) helps to induce LL‐37 expression [55, 56]. Another important inducer of cathelicidin expression in humans, primates, and rodents is butyrate [57, 58, 59, 60], a short‐chain fatty acid produced by gut microbiome bacteria [61, 62]. Phenylbutyrate is also active in inducing LL‐37 [63, 64], acting in synergy with vitamin D3 [65, 66]. Notably, the cathelicidin LL‐37 and other antimicrobial peptides are strongly upregulated in humans by exercise, as measured in saliva [67].

**Fig. 4:**
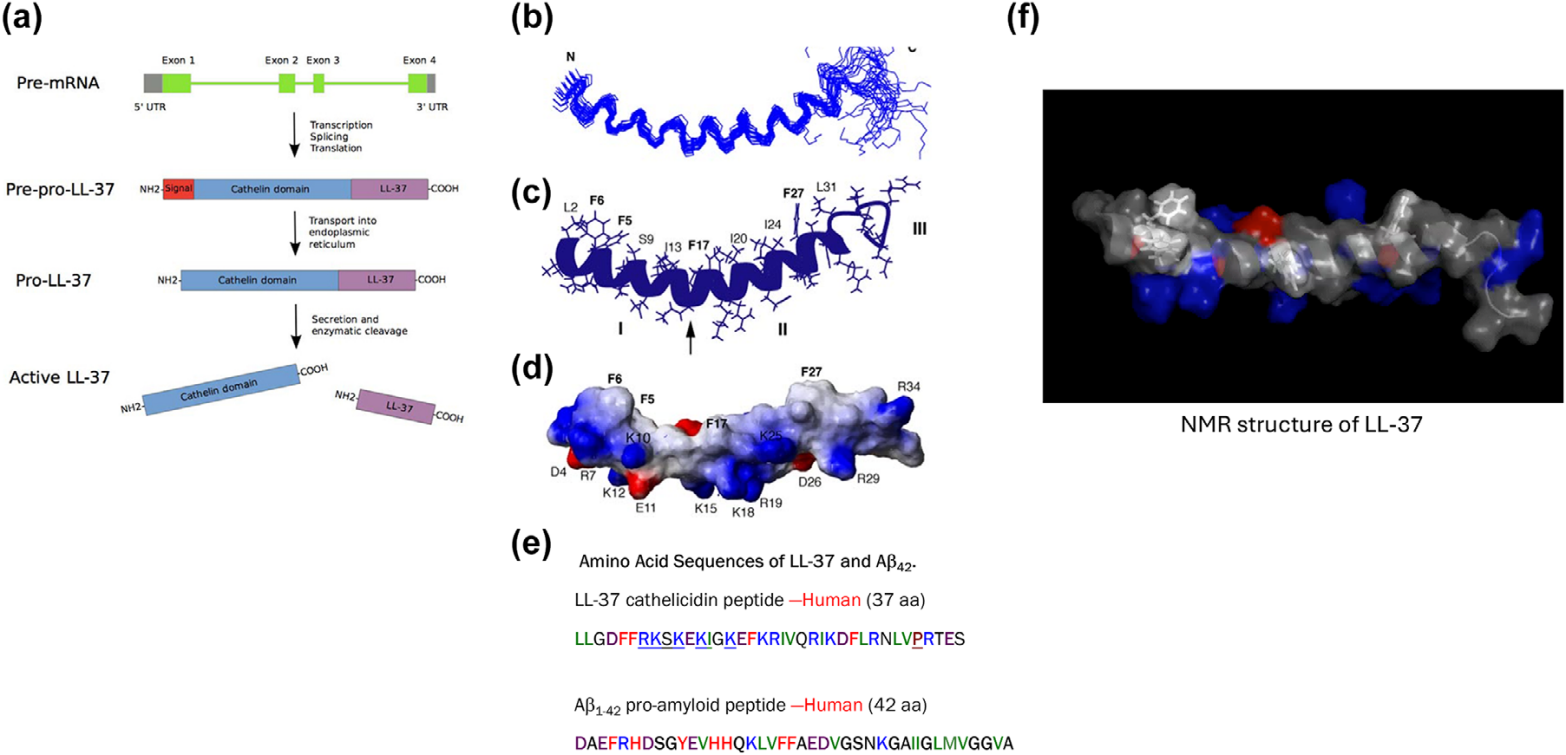
**LL‐37 expression and structure comparison to Aβ**. (a) Schematic of the CAMP gene and LL‐37 processing. (b) Ensemble of 28 backbone structures with residues 2–30 superimposed. (c) Ribbon representation of LL‐37 structure. (d) Potential surface of LL‐37 with the hydrophobic surface at the top and hydrophilic surface at the bottom. (e) Comparison of amino acid sequences of LL‐37 and Aβ42. (f) NMR structure of the human cathelicidin peptide, LL‐37. Source: Adapted from [68, 168].

The three‐dimensional folded structure of human cathelicidin LL‐37 peptide in association with anionic micelles has been determined by 2D‐NMR (Fig. 4) [68]. It is a helical peptide composed of 37 amino acids (including six arginines and five lysines), which carries +6 electrostatic charge at physiological pH. As the factotum human host defense molecule, LL‐37 is a natural, endogenous broad‐spectrum antibacterial, antiviral, antifungal, and antiparasitic peptide “drug” [49]. LL‐37 directly kills a broad spectrum of Gram‐negative and Gram‐positive bacteria [49, 69] as well as fungi [70] and inactivates viruses including HSV‐1, influenza A, HIV, hepatitis C, Kaposi's sarcoma human herpesvirus, Epstein–Barr virus, and vaccinia virus, among others [71, 72], utilizing a complex combination of membrane‐disruptive and intracellular nucleic acid‐ and ribosome‐targeting biophysical mechanisms [69]. Further, LL‐37 can prevent the formation of bacterial biofilms by binding to structural proteins that stabilize the biofilm matrix [73]. To date, the LL‐37 peptide is known to be utilized by and deployed from a myriad of human cell types, including neutrophils, macrophages, microglia, T cells, NK cells, dendritic cells, platelets, basophils, eosinophils, neurons, and mast cells, as well as by all types of epithelial and endothelial cells [49, 52]. Active LL‐37 peptide is released from its pre‐pro‐protein, hCAP‐18, through the action of a unique, dedicated enzyme (Proteinase 3), and in vivo, is released in the presence of pathogens within a response time of about 3–5 min [49, 74].

LL‐37 is highly effective at repelling these myriad types of pathogens and in preventing infections at barrier layers [74], such as those of epithelia and mucosa. Finally, and importantly, LL‐37 is strictly necessary and is a direct molecular participant in the cellular processes of both autophagy [64, 75, 76] and macroautophagy [77].

**Fig. 5:**
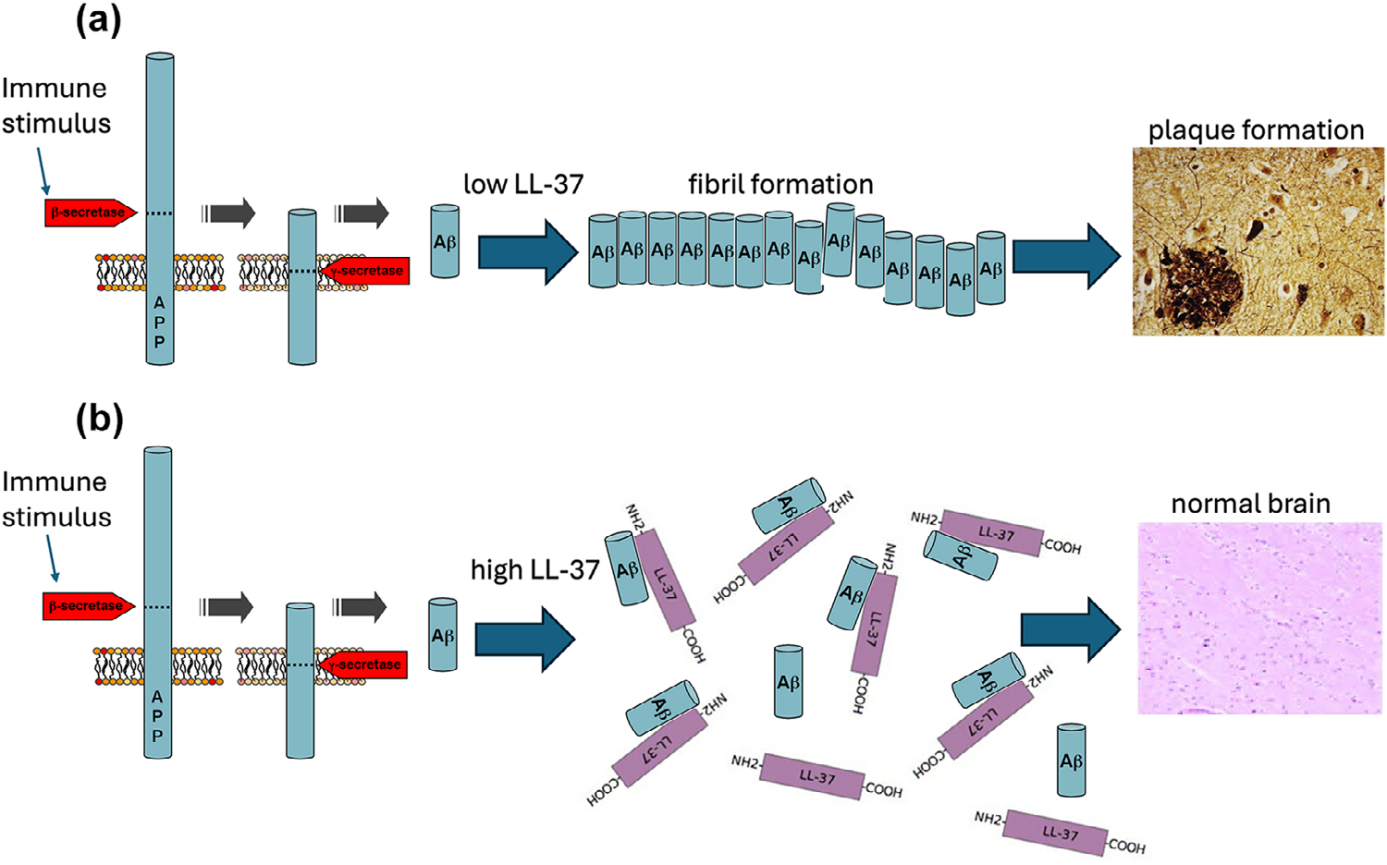
**LL‐37 binding to Aβ inhibits fibril formation**. (a) Low LL‐37 levels may lead to plaque formation, whereas (b) high LL‐37 levels inhibit fibril formation. Source: Adapted from [169, 170, 171]. Image of AD brain pathology reprinted from [171] with permission from Elsevier.

LL‐37 is mainly expressed in epithelial cells and the immune system, with high levels occurring in the gastrointestinal tract [78] and neutrophilic granulocytes. It was found to be upregulated in the lungs of patients who died with pneumonia [78] and may itself play a role in neuroinflammation (as observed in in vitro microglia/neuron cell culture experiments) [78]. In 2017, it was shown by a host of molecular biophysical methods that LL‐37 peptide strongly inhibits Aβ fibril assembly [1]. LL‐37 binds to the Aβ peptide with sequence‐specificity. LL‐37 binds more strongly to small Aβ oligomers than mature Aβ fibrils and thus tends to *prevent* the assembly of Aβ oligomers into larger fibrils and plaques. LL‐37 binding to Aβ in vitro completely prevents the formation of β‐type secondary structure by Aβ [1]. Neuron cell culture studies using primary human microglia isolated freshly from human brains showed that binding and complexation of LL‐37 with Aβ protects neurons to a great degree from Aβ’s microglia‐mediated neurotoxicity and neuroinflammation [1]. More recent data demonstrate that the relative molar ratios of LL‐37 levels modulate the *rate* of Aβ fibril formation (Fig. 5), where an equimolar (1:1) Aβ:LL‐37 ratio completely prevents Aβ oligomerization and fibril formation, and lower molar ratios (e.g., 2:1, 4:1, 8:1, and 16:1 ratios of Aβ:LL‐37) slow Aβ fibril formation. Interestingly, LL‐37 has also been shown in vitro to bind to and inhibit fibril formation for not only Aβ, but also for other amyloidogenic peptides and proteins, including IAPP, α‐synuclein, and bacterial biofilm Curli Fimbriae [73, 79, 80].

Finally, the endogenous expression of the unique human host defense peptide LL‐37—which is integral to both autophagy and macroautophagy and prevents the formation of Aβ oligomers, fibrils, and plaques—is lower in females than in males [81, 82], for reasons that are not yet understood. Importantly, this finding may help to explain (among many other possible factors) why women have a substantially higher risk of AD, where AD affects two‐thirds of women, but only one‐third of men by the end of their lives [83].

### Effect of citrullination of LL‐37

Citrullination of LL‐37 blocks both its antimicrobial and immunomodulatory activities [84] and completely inactivates its pleiotropic functions in vivo [85] (this may also be true of ApoE‐ε4/ε3). For example, LL‐37 is physically essential for autophagic degradation of pathogens in monocytes and macrophages. LL‐37 contributes to the autophagic clearance of *Pg* from human keratinocytes [86]. Moreover, LL‐37 is normally active against herpesviruses [87], blocking their entry into host cells [88]. Citrullination of TNF‐α by PPAD enzymes also blunts its immunostimulatory function, reducing the ability of TNF‐α to stimulate inflammatory cytokine production [89]. Taken together, *Pg*’s gingipains and PPAD are likely to greatly reduce local host defense responses in the vicinity of *Pg* colonies, or where the gingipain enzymes are found (they can also be transported in extracellular vesicles, EVs, released from *Pg*‐infected host cells).

Recently, it was suggested that PPAD citrullination may be a factor in the pathogenesis of AD [90]. Indeed, citrullinated proteins are abnormally accumulated in hippocampal extract from the brains of patients with AD [48].

### Role of neuroinflammation and the innate immune system

Neuroinflammation is a key hallmark of late‐onset AD [32, 91, 92, 93]. We hypothesize that neuroinflammation is not solely a consequence of Aβ accumulation (as posited by the conventional amyloid cascade paradigm) but is instead coincident with a fundamental cause for Aβ deposition (likely to be polymicrobial infection, e.g., *Pg* and a herpesvirus such as HCMV or HSV‐1). This infectious origin hypothesis therefore posits pathogens as the source of molecules that induce neuroinflammation (e.g., an LPS, or a viral surface protein) and, potentially as well, a pathogenic source for virulence factors that dysregulate innate immune responses and host defense [94]. In this case, Aβ is not simply a “harmful” molecule that aggregates to form plaques but also constitutes a basic element of innate immune defense and is thus also a *beneficial* molecule [95].

Ultimately, as reactivations of chronic infections [96] occur and the chronic production of Aβ increases, its antimicrobial effect may be blunted by loss of active Aβ through recruitment to plaque formation; therefore, neuroinflammation becomes chronic, and ultimately Aβ deposition proceeds and results in senile plaque formation [15]. The deposition of plaque may be an initiator of inflammatory processes that finally lead to the destruction of neighboring neurons; or alternatively and/or additionally, aspects or products of chronic herpesvirus infections may also cause neuroinflammation and neurodegeneration, including phagocytosis of damaged neurons.

It is known that exosomes/microvesicles—related to a “Senescence‐Associated Secretory Phenotype” (SASP) [97] and others not related to SASP—facilitate intercellular communication and modify immune responses by transporting miRNAs, mRNAs, and proteins through bodily fluids [98]; however, they may be highly tissue‐specific in their content and function. Their inflammation‐modulating role was extensively studied in cancer [99]. Exosomes from *Pg*‐affected periodontal ligament stem cells were shown to alter T‐cell phenotypes [100]. Six particular candidate microRNAs were identified as biomarkers of aging in periodontitis [101]. Studies also involved exosomes in AD as a means to propagate Aβ pathology, neuroinflammation, and oxidative stress [102, 103, 104, 105, 106] across the BBB [107]. Nonetheless, despite abundant evidence demonstrating a role for exosomes in regulating inflammatory responses, the exact mechanisms remain unclear. Therefore, an improved understanding of the role of exosomes in inflammation at different stages of human AD may assist in its prevention and treatment.

### The role of biofilms in late‐onset AD

Late‐onset AD is almost certainly not induced by a short‐lived microbial infection. Because it evolves and progresses over years, the infection(s) at the origin of increased Aβ synthesis must also be chronic. Chronic bacterial infections are almost invariably found as biofilms, a community of organisms protected by an extracellular matrix that ensures their persistence. Many biofilms also exude amyloid that may in the case of AD contribute to senile plaque formation [108], some of which directly inactivate host defense peptides such as LL‐37 [73]. Because of the long incubation time in AD, these biofilms and their products must be present several years before clinical appearance of AD, and thus they may be detectable in the blood. However, how they could induce Aβ synthesis by neurons or inhibit its clearance by glial cells remains to be elucidated. They might secrete products that enter the bloodstream. Products encapsulated in exosomes or extracellular vesicles cross the BBB more easily, especially in later life when the BBB may be weaker; might such products react with neuronal TLR receptors to induce inflammation and/or Aβ synthesis?

Microorganisms in the mouth, especially *Pg*, no doubt survive for long periods with the help of biofilms that they and other microorganisms produce. Biofilms and pathogens from the oral microbiome could have direct access to the brain through the lingual or olfactory bulb (trigeminal nerves) or from the gut via the vagal nerve. Indeed, the sense of smell is often reduced in patients with AD [109]. Mice infected intravenously with the fungus *Ca* show mild memory impairment that resolves with fungal clearance, whereas it was found that Aβ peptide accumulates on the fungal cells within fungal‐induced glial granulomas [110]. If delivered by oral gavage, *Ca* is able to robustly colonize the mouse GI tract and persist for at least 2 months [111]. *Pg* is also known to form *dual biofilms* with the fungus *Ca*, which promotes the survival of *Pg* by reducing oxygen tension (*Ca* is highly aerobic), as well as enhancing biofilm adherence and *Pg*’s virulence [112, 113]. It was hypothesized that the formation of a dual biofilm with *Ca* may be very important to *Pg*’s survival in the oral cavity. Dual *Pg*/*Ca* co‐species biofilms synergize in the lung to weaken the lung's epithelial barrier [114]. In a Japanese study of 86 *Pg*‐positive periodontitis patients (mean age 70.4 years), *Ca* was detected in 22 (25.6%) of the 86 patients, with the more elderly patients in their 80s having a higher probability of having dual *Pg*/*Ca* biofilms [115]. This role of *Ca* in supporting the chronic survival of *Pg* in dual biofilms and in increasing *Pg*’s virulence offers a novel possible explanation for why low‐carbohydrate “ketogenic” diets may have a salutary effect on mental health [116, 117]. The ketogenic diet is also being explored as a potential treatment for the negative systemic effects of AD and holds promise [118].

### Senile plaques

Plaques in the brain of AD patients and even plaques in non‐ or pre‐AD patients have many properties in common with biofilms [119, 120, 121]. Biofilms arise from the aggregation of substances such as polysaccharides, proteins, and nucleic acids that are secreted by individual or colonies of microorganisms. The exact composition of the biofilm varies according to the type of microorganism and surrounding tissue. Biofilms insulate microorganisms from their environment, notably preventing both immune and antimicrobial destruction. Within the biofilm, microorganisms can communicate and share nutrients and release virulence factors that are protective against the immune response. Thus, considering the infectious hypothesis and the antimicrobial role of Aβ, it was suggested that plaques found in the brain could be Aβ‐structured or inclusive biofilms that allow the survival of the various pathogens but also contain them. This was shown for *Treponema* [119] and for *Chlamydophila pneumoniae* [122]. The presence of such plaques, in the absence of dementia, could mean that the cohesive power of these biofilms has contained the pathogens, effectively inactivating them or greatly slowing their growth, so that no clinical manifestations have appeared. However, periodic release of microorganisms from the biofilms could give rise to reactivations of the infections, eventually provoking clinical symptoms.

### *Pg* as instigator

We propose that late‐onset AD may emerge due to involvement of *Pg* as an initial instigator, potentially supported by *Ca* in dual biofilms, followed by a combination of factors, including other microorganisms, especially viral infections by endemic herpesviruses, including HCMV and HSV‐1 and VZV, and subsequent innate immune system dysfunction resulting in a loss of protection against further infection, inflammation, and weakening of the BBB. A CD83(+) microglial subtype (characterized by its dual role in controlling and resolving neuroinflammation, rather than simply indicating an activated, pro‐inflammatory state) was identified in 47% of brains from patients with AD versus 25% of unaffected controls [123], and this subtype is significantly associated with elevated immunoglobulin IgG4 and HCMV [124]. VZV infections can also cause the reactivation of HSV‐1 infections, a process correlated with AD onset [125, 126]. These factors are associated/reinforced with aging but may also be promoted by the presence of *Pg* in the oral cavity, blood, brain, or immune cells. This theory is consistent with the observation that the virulence factors produced by *Pg* can inactivate and dysregulate the innate immune response—in particular, greatly downregulating the critical antiviral responses provided by the IFN‐λ pathway [37]. If *Pg*‐induced innate immune system dysregulation indeed plays such a role in cases of late‐onset AD, then one approach to the prevention or treatment of AD may lie with the use of antibiotics that target *Pg* (e.g., metronidazole can be used together with amoxicillin to kill anaerobic oral bacteria, including *Pg*), or with other pharmaceuticals that target *Pg* [127], prevent *Pg* from entering the brain or nerves, or inactivate *Pg*’s toxic virulence factors, possibly combined with antiviral drugs that can prevent or reduce the replication of herpesviruses. Alternatively, the drastic reduction of carbohydrates in the diet may be important for the prevention of recurrence, if indeed *Ca* is critical to *Pg*’s survival within dual biofilms, as much research has shown. Once the true etiology of late‐onset AD is understood to be infectious in nature, both effective prevention and remedies are in reach, likely at a reasonable cost.

### Persistence as biofilms

We hypothesize that chronic *Pg* infection—persisting as biofilms—stimulates Aβ synthesis, weakens immune defenses (particularly innate immunity via *Pg* virulence factors), and promotes chronic inflammation, ultimately leading to late‐onset AD. Aging may simultaneously contribute to waning immune defenses and chronic inflammation. Chronic periodontitis caused by *Pg* infection generally lasts for many years, if not for a lifetime, after *Pg* has colonized the oral cavity [128]. Such long‐term survival requires the formation of biofilms. *Pg* was shown to exist within very particular spatial structures within the oral microbiome, research that provided fascinating images [129]. Periodontitis is also associated with an increase in cognitive decline in AD, independent of baseline cognitive state, which may be mediated through its effects on systemic inflammation [34]. Experimental evidence exists that senile plaques may enclose microorganisms, cellular debris, microbial DNA, and amyloids [130].

Biofilms may also be involved in other human chronic diseases, such as atherosclerosis, thus obfuscating their role in AD. However, it is likely that the infectious signature of chronic infections in the brain will be different from those in blood vessels or elsewhere, because the immune system is not nearly as efficient in the brain as compared with the periphery.

### Chronic oral infection with *Pg* results in an Aβ response in the brain

Initially, two research groups [36, 40] demonstrated that chronic oral infection of wild‐type mice with *Pg* leads to induction of Aβ in the brain. Oral *Pg* infection was also observed to cause Tau phosphorylation [40]. These were seminal findings, because the vast majority of murine research in AD has used transgenic mice that overexpress human Aβ peptides behind a neuron‐specific Thy1 promoter. In those models, AD could be largely prevented or treated by reducing Aβ expression or promoting its rapid clearance. However, these findings did not translate very well to humans when antibody drugs against Aβ were tested [7], even those with familial mutations and overexpression of Aβ. It was a groundbreaking discovery that AD‐like pathology can be produced in wild‐type mice via *Pg* infection.

The approaches used by the two groups were significantly different: Ilievski et al. used young (8‐week‐old) C57BL/6 mice, with repeated oral application of *Pg* W83 for 22 weeks (MWF each week) [40], whereas Dominy et al. [36] used aged (44‐week‐old) BALB/c mice, with oral infection every other day for 6 weeks with *Pg* W83 (as well as two control groups that were infected on the same schedule with *Pg* mutants: a Kgp knockout (DKgp) [Dkgp (602‐1732) Emr] [131] or RgpA RgpB double knockout (DRgp) (DrgpA rgpBD495‐B Cmr, Emr) [132]). In the much longer‐term (22‐week) Ilievski study [40], chronic W83 *Pg* infection caused neuroinflammation, neurodegeneration, microgliosis, astrogliosis, and the formation of intra‐ and extracellular Aβ and neurofibrillary tangles, all key pathognomonic signs of AD. The two studies are both important because they demonstrate in two different strains of wild‐type mice, both young and aged, that *Pg* and/or its virulence factors can be transported from the oral cavity to the brain, potentially via the trigeminal nerve [133] and/or the gut–brain axis [134], the consequences of which are consistent with the physical signatures of late‐onset AD, which have been replicated now in multiple studies [134, 135, 136]. Rodent studies have also demonstrated impaired cognition and behavior with *Pg* infection or with the oral administration of *Pg* outer membrane vesicles [134, 135, 137, 138].

### Dysregulation of LL‐37 compromises the BBB and endothelial and epithelial barrier tissues

Due to the significant role LL‐37 plays in the immune system, the effects of its dysregulation and degradation by *Pg* virulence factors are considerable and systemic and could help explain the pathology of AD. These effects extend beyond the function of LL‐37 as an antimicrobial peptide. One example is the effect that the dysregulation of LL‐37 can have on tight junction (TJ) proteins. These proteins are found in various barrier layers throughout the body, including the BBB.

TJ proteins are situated at the TJs of epithelial, endothelial, and myelinated cells [139]. They are critical to the formation and proper functioning of various biological barriers in the human body, including the BBB, gut epithelia, vascular endothelia, and epidermal keratinocytes [140]. One of the primary functions of the BBB is its strict regulation of paracellular permeability due to the presence of junctional complexes (tight, adherens, and gap junctions) between the endothelial cells [141]. TJ protein complexes in the BBB maintain low paracellular permeability by sealing the paracellular route between opposing brain microvascular endothelial cells [142]. Alterations in junction assembly and function significantly affect BBB properties, particularly barrier permeability [141]. Thus, the expression and regulation of TJ proteins are crucial to the proper functioning of the BBB.

LL‐37 upregulates TJ proteins (including several claudins and occludin) in endothelial cells [50], enhances the membrane distribution of these proteins, and directly modulates endothelial stiffness and barrier permeability [51]. Because of this relationship between LL‐37, TJ protein expression, and TJ barrier properties, the maintenance of basal expression levels of LL‐37 is crucial to the proper functioning of the BBB and of other barrier layers throughout the body. It is thus possible that chronic dysregulation of LL‐37 may contribute to the development of AD through degradation of the BBB.

Degradation of the BBB has been tied to AD and other neurodegenerative disorders. In particular, BBB disruption allows influx into the brain of neurotoxic blood‐derived debris, cells, and microbial pathogens and is associated with inflammatory and immune responses, which can initiate multiple pathways of neurodegeneration [143]. BBB breakdown has been demonstrated by neuroimaging studies in the living human brain, postmortem tissue, and biomarker studies in various neurodegenerative disorders, including AD and dementia [143]. Similarly, increased BBB permeability has been correlated to mild cognitive impairment, suggesting that BBB breakdown may actually precede neurodegeneration [143]. This concept is supported by data from experimental models of BBB breakdown, which causes neurodegenerative changes over time, and studies in patients with early AD confirmed BBB breakdown in several grey matter and white matter regions [144]. Indeed, dysfunction of claudin‐5, the most enriched TJ protein, has been implicated in AD and other neurodegenerative disorders [145].

The significance of the effect of LL‐37 on TJ barriers, and especially the BBB, is further illustrated by the pathogenic response of the BBB. Thus, for example, LL‐37 (as well as defensins) is expressed in the epithelial cells of the BBB in response to the presence in the body of bacterial infections such as meningitis [146] and thus plays an important role in the innate immune response against pathogens in the central nervous system [147].

### The BCG vaccine strongly upregulates LL‐37—potential implications for AD

One innovative approach to broad upregulation of innate immunity was previously discussed by Aloul et al. [148] and is through intake of oral inducers of LL‐37, such as vitamin D, butyrate (or phenylbutyrate), curcumin, stilbenoids, or genistein, or through exercise or exposure to UVB light [148]. A second innovative approach to broad upregulation of innate immunity may be with the use of the Bacillus Calmette–Guérin (BCG) vaccine. Although this vaccine is not currently used broadly in the USA (as it would abrogate the currently used TB test), it is still given to the citizens of many other countries, such as in Japan [149]. It has recently been proposed that re‐introducing early BCG vaccination in Europe and introducing it for the first time in North America may help to prevent AD by upregulating innate immunity [149]. It has been shown, in an APP/PS1 mouse model of AD, that early BCG vaccination essentially prevented AD‐like disease [150]. That study used 4Aβ1‐15 vaccination as treatment control [150]. Note, though, that this BCG study [150] did not cite the fact that BCG vaccination upregulates cathelicidin in the gut [151], in addition to providing broad, nonspecific innate immune protection against infection by virtually any bacterial, viral, or fungal pathogen [152, 153, 154, 155]. A recent, comprehensive mouse study showed specifically that IV BCG enhances antiviral immunity in particular [156]. Neonatal BCG vaccination is neuroprotective and enhances neurogenesis and synaptic plasticity in mice [157, 158, 159]. Indeed, the repeated intravesicular instillation of the BCG vaccine into the bladder, for the treatment of bladder cancer, has been shown to produce a four‐fold lower risk of those BCG‐treated patients developing AD later in their lives (a 58% relative risk reduction in developing AD within 3–7 years) [149, 160, 161]. Whether this effect is mediated via increased expression of LL‐37, by the upregulation of antiviral immunity (which LL‐37 may also provide), or by a general immune‐activating mechanism remains to be revealed [151, 162].

### The shingles vaccine is protective against dementia

Recent studies have shown that the shingles (herpes zoster) vaccine is associated with an ∼20% lower relative risk of dementia [163, 164]. This protective effect was observed for both the live‐attenuated vaccine and the recombinant vaccine, and, interestingly, this effect was stronger among women than men. Although it has yet to be measured directly, the immune response to the virus triggered by the vaccine is likely stimulating production of LL‐37 as a secondary effect, which could have relevance for the hypothesis presented here.

### Which knowledge gaps remain?

Even though the link between chronic immune activation and AD development appears strong, there remain important gaps in our knowledge. First, the causal relationship between infection, inflammation, and the development of AD is not yet fully elucidated. In fact, it is possible that reverse causality is involved, that is, that amyloid plaque formation causes inflammation and not vice versa. (Certainly, infections cause inflammation; and Aβ oligomers are directly neuroinflammatory.) However, the evidence from two independent experiments in mice indeed support a direct causal role for *Pg* in plaque formation [36, 40]. Although these data are convincing, they are performed in well‐controlled conditions in an animal model, which is far from the situation in humans. Second, the temporal aspects of AD pathogenesis need to be clarified. AD develops over decades, and the detailed aspects of how a bacterial/fungal or viral infection or inflammatory process can trigger and sustain this process are not resolved. One possibility is that *Pg*—which can colonize the gums of susceptible individuals for long periods of time—causes a kind of secondary immunosuppression by degrading or modifying important innate effector molecules, such as interferons and the cathelicidin peptide LL‐37, the latter of which can also bind to Aβ and prevent fibril formation [1]. The precise mechanisms for how this process, which occurs in the oral cavity and to a lesser extent systemically, can impact the brain and cause AD remain elusive. Third, the lack of proper models in animals hampers the possibility to obtain results with human relevance. In line with this problem, there is a lack of human interventional trials where treatment of infection or inflammation has had a major impact on AD pathogenesis. The best evidence is probably from studies where vaccination has been studied in relation to the risk for AD. Notably, both BCG vaccination and varicella vaccination have been shown to reduce the risk for AD. The mechanism could involve specific effects against certain pathogens, but also a general immune response elicited by vaccination has been suggested. To solidify this hypothesis for a bacterial/viral coinfection cause of the inflammation known to prevail in AD's pathogenesis, further research is needed, including prospective clinical trials specifically targeting *Pg* infection, because this is proposed to be the main instigator of this disease process. One interesting approach is a study testing whether a gingipain inhibitor can have beneficial effects on the cognitive functions of patients with AD [165, 166]. The results, whether negative or positive, will shed important light on the role of *Pg* in AD pathogenesis. Hence, the field needs more of these studies with a prospective and interventional design. However, the intervention should probably start early, long before any AD pathology has occurred, which poses logistical and ethical challenges.

In summary, this perspective proposes a novel hypothesis that unifies the amyloid cascade and infectious theories of AD through the lens of chronic innate immune system dysregulation, with a focus on the central role of chronic oral and gut *Pg/*herpesvirus coinfection, LL‐37 dysregulation, ApoE degradation, and resultant immune dysfunction. We summarize the key evidence supporting this hypothesis in Table 1. By highlighting the pivotal role of *Pg* and its gingipain virulence factors in the degradation and inactivation of key innate immune proteins, we suggest a mechanism by which a persistent, low‐level infectious assault on the brain, especially by unleashed herpesviruses that *Pg* gingipains allow to replicate by suppression of IFN‐λ, can trigger the cascade of events leading to Aβ plaque accumulation, Tau phosphorylation, neuroinflammation, and ultimately, neurodegeneration. This perspective not only offers a comprehensive framework for understanding AD pathology but also suggests promising avenues for prevention and intervention, including lifestyle modifications, upregulation of innate immune defenses by early BCG vaccination and oral dosing with vitamin D3 and other LL‐37 inducers, other vaccination strategies (e.g., use of the shingles vaccine or the BCG vaccine for older individuals), and targeted antimicrobial therapies, including both antibiotics against *Pg* and gingipain inhibitors. Future research should focus on validating these proposed mechanisms and exploring the clinical efficacy of these preventative and therapeutic approaches in mitigating the progression of AD (summarized in Table 2). Although many knowledge gaps remain, the NIH's recent funding (USD 49.2 million) of a Phase 2 clinical trial of gingipain inhibitors to treat AD is an encouraging sign that this hypothesis is now a serious contender among the potential causes of dementia [165, 166].

**Table 1:** Evidence to support the hypothesis of innate immune system dysregulation as a potential cause of Alzheimer's disease using Porphyromonas gingivalis as a focus.

Role of *Pg* as a key upstream event in the initiation and progression of periodontal disease	Role of *Pg*, a key oral pathobiont, and its gingipain and PPAD enzymes, as a key component in innate immune system dysregulation and AD
Association studies link *Pg* to Alzheimer's disease [33, 36]	Breakdown of epithelial barriers, including the BBB, promote microbial invasion into the brain through degradation of tight junctions [50, 138]
Mechanistic studies demonstrate *Pg*’s role in formation of amyloid plaques, tau tangles, neuroinflammation, and translocation of pathogens to the brain [36, 40]	Invasion into the brain of *Pg* enzymes and toxins via exosomes and membrane coated vesicles [102, 103, 104, 105, 106, 107, 134, 135, 137, 138]
Clinical trials with targeted antimicrobials show improvements in both the clinical and pathophysiological markers of AD [36]	Enhanced invasion, survival and pathogenic activity of *Pg* through reciprocal interactions with oral *Candida albicans* [112, 113, 114]
Beneficial interactions between *Pg* and viruses such as HSV‐1, VZV, and HCMV may enhance key upstream events in immunity dysregulation and AD [38, 113, 125]	Down regulation of antiviral activity, including interferon and TNF‐α [37, 38, 89]
	Deactivation and degradation of antimicrobials through its PPAD enzyme through citrullination and/or degradation (e.g., ApoE3, ApoE4, LL‐37, and complement) [44, 45, 46, 47, 48]
	Direct and indirect effects of deactivation of LL‐37 on immunity dysregulation [84, 85, 86, 87, 88, 89] Reduced anti‐biofilm formation activity [73] Increased Aβ oligomerization and fibril formation [1]

Abbreviations: AD, Alzheimer's disease; ApoE, Apolipoprotein E; BBB, blood–brain barrier; HCMV, human cytomegalovirus; *Pg, Porphyromonas gingivalis;* PPAD, peptidylarginine deiminase.

**Table 2:** Knowledge gaps and proposed future research focused on the hypothesis of innate immune system dysregulation as a potential cause of Alzheimer's disease.

1. Examine the full range of interactions between key pathogens in three domains (i.e., *Pg*, *Ca*, HSV) and identify which interactions are critical for dysregulation of innate immunity
2. Establish a time course and progression of microbial, inflammatory, immune and pathophysiological events (linear or circular) for the initiation and progression of AD
3. Identify and then translate the optimal combinations of antimicrobials and anti‐inflammatory agents to clinical trials to assess their efficacy and safety on reducing dysregulation of innate immunity and AD symptoms and pathophysiology
4. Study the underlying mechanisms of certain antibacterial and antiviral vaccines such as BCG and varicella on reducing dysregulation of innate immunity and AD
5. Conduct clinical and mechanistic nutritional studies to assess the potential beneficial effects of specific diet regimens such as ketogenic diet on reducing dysregulation of innate immunity and clinical and pathophysiological markers of AD

Abbreviations: AD, Alzheimer's disease; *Pg, Porphyromonas gingivalis*.

## Conflict of interest statement

The authors declare no conflicts of interest.

## Data Availability

Data sharing is not applicable to this article as no datasets were generated or analyzed during the current study.
